# Pterostilbene attenuates intestinal epithelial barrier loss induced by high loading intensity of exercise

**DOI:** 10.3389/fnut.2022.965180

**Published:** 2022-08-04

**Authors:** Lidong Zhang, Guoliang Tian, Li Huang, Min Zhou, Jundong Zhu, Long Yi, Mantian Mi

**Affiliations:** Chongqing Key Laboratory of Nutrition and Food Safety, Research Center for Nutrition and Food Safety, Chongqing Medical Nutrition Research Center, Institute of Military Preventive Medicine, Army Medical University (Third Military Medical University), Chongqing, China

**Keywords:** exercise, pterostilbene (PTE), lipopolysaccharide (LPS), intestinal barrier, intestinal flora

## Abstract

Mounting evidence suggested that high loading intensity of exercise might be detrimental to human health, especially the gastrointestinal tract. Pterostilbene (PTE), derived from grapes and blueberries, might reach a high concentration of intestinal contents. Our study aimed to evaluate PTE’s ability to prevent the loss of intestinal epithelial barrier in high loading intensity of exercise. The exercise model was established by the forced running of mice. An effective HPLC-UV method was developed to quantify PTE concentration in intestinal content. The mRNA changes were detected by quantitative polymerase chain reaction (qPCR). The structure of intestinal flora was analyzed by 16S rRNA sequencing. The PTE (100 mg/kg/d) could significantly attenuate exercise-induced intestinal epithelial barrier loss. Moreover, the HPLC-UV assay showed that the PTE concentration of intestinal content could last 12 h. Furthermore, the exercise increased the abundance of Alistipes, which was related to lipopolysaccharide (LPS) production but could not be reversed by PTE intervention. Besides, cell experiments showed that PTE could promote the expression of intestinal epithelial tight junction (TJ) molecules *in vitro*. In conclusion, PTE has a significant interest in preventing exercise-induced intestinal damage.

## Introduction

It is a known fact that moderate exercise can prevent and cure various metabolic diseases and enhance immunity (1–3). Otherwise, the sustained high loading intensity of exercise, which exceeds 60% maximal oxygen uptake (VO_2_ max) or 70% of maximum heart rate reserve, such as a 50-km forced march, marathon, or triathlon, may cause many health hazards to the body (4, 5). High loading intensity of exercise causes metabolic disorder of skeletal muscle, leading to repetitive tissue micro-trauma of muscle, connective tissue, bone structure, and chronic inflammation (6). In severe cases, some studies show that 86% of athletes have severe gastrointestinal syndrome (GIS) (7).

The intestinal flora plays an essential role in host physiology and health. Mounting evidence suggests that exercise could alter the structure of intestinal flora (8, 9) and is a crucial modulator of intestinal flora (10). Besides, recent evidence shows that stress during exercise is highly related to the changes in the intestinal flora (11). However, there is no direct evidence that high loading intensity of exercise causes dysbiosis of intestinal flora. The effect of high loading intensity of exercise on intestinal flora is still unknown. Therefore, our study aimed to explore the effect of high loading intensity of exercise on intestinal flora.

The intestinal barrier has become a focus of biomedical research, divided into chemical, mechanical, biological, and immune barriers according to their functions (12, 13). The mechanical barrier is composed of occludin, claudins, and zonula occluden (14, 15), which could prevent lipopolysaccharide (LPS) from transferring into serum (13). The LPS could cause harmful inflammatory reactions. LPS could cause TJ dysfunction through the activation of the immune system or the inflammatory process, closely associated with the initiation or development of intestinal diseases (16). Besides, LPS could cause altered membrane permeability, through the disruption or relocation of tight junction (TJ) proteins, following redox-sensitive mitogen-activated protein kinases (MAPKs) modulation (17). A growing body of research indicates that high loading intensity of exercise could cause intestinal epithelial barrier damage (18–20). As for athletes, the sustained high loading intensity of exercise training is frequent (7), and barrier integrity is vital for athletes to prevent LPS from transferring into the serum to avoid GIS. Therefore, a new strategy for preventing intestinal barrier damage from high loading intensity of exercise is required for athletes.

Given the crucial role of the practical application for athletes, dietary approaches are preferentially recommended to prevent intestinal barrier loss (21). In this sense, natural bioactive substances such as plant polyphenols are emerging as sports nutrition supplements for GIS prevention (22, 23). Research studies have recently indicated that a Mediterranean diet containing a high proportion of polyphenolic compounds (24) could significantly prevent intestinal barrier dysfunction (25). The phenolic compounds such as pterostilbene (PTE) are widely reported and rooted in blueberries and grapes (26). Mounting evidence has further proved that PTE and its metabolites could improve the alteration in epithelial permeability induced by LPS (17). PTE might slow LPS induced transepithelial electrical resistance decrease, preserve TJ proteins levels, and reduce MAPK phosphorylation to attenuate alteration of epithelial permeability. Furthermore, some studies find that PTE protects the intestinal epithelial barrier through the NF-κBMLCK/p-MLC signal pathway in mice (27). Besides, PTE could attenuate oxidative stress-induced intestinal injury by improving mitochondrial redox homeostasis (28). However, the protective role of PTE in exercise-induced intestinal barrier damage remains unclear.

In this study, we attempted to investigate the effect of PTE against damage induced by high loading intensity of exercise. Our findings revealed that exercise caused the intestinal epithelial barrier loss by altering intestinal flora’s structure. Otherwise, the intervention of PTE (100 mg/kg/d) could promote the expression of intestinal epithelial TJ molecules to prevent intestinal damage.

## Materials and methods

### Chemicals and reagents

The LPS and 4′,6-diamidino-2-phenylindole (DAPI) were purchased from Sigma (St. Louis, MO, United States). PTE (≥ 98% purity analyzed by HPLC) was obtained from Chengdu Must Bio-Technology. Occludin antibodies were purchased from Proteintech (Chicago, United States). The 3,5,4′,-trimethoxy-trans-stilbene (TMS) was obtained from Solarbio (Beijing, China). The penicillin–streptomycin was purchased from Beyotime (Shanghai, China). The fetal bovine serum was obtained from HyClone (Logan, UT, United States).

### Experimental animals and design

**FIGURE 1:**
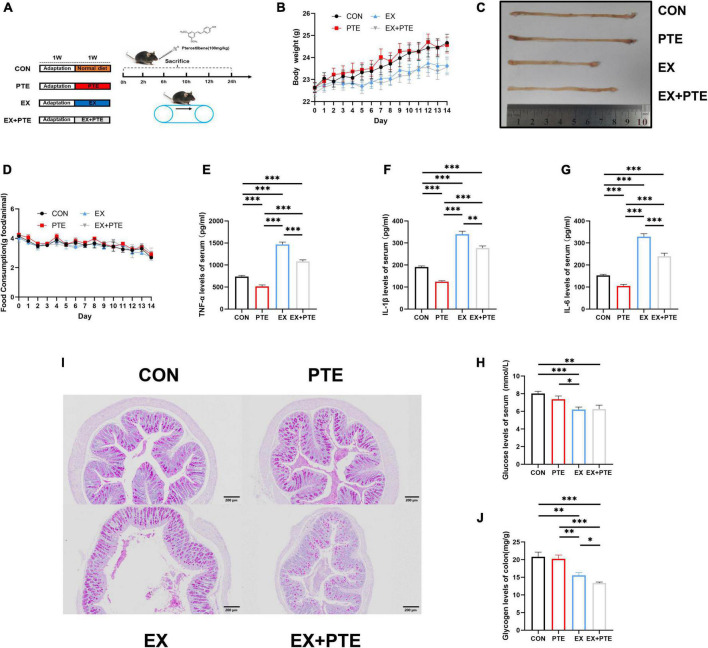
Exercise-induced intestinal barrier disruption and inflammation in C57BL/6 mice. **(A)** Part1: mice gavaged with pterostilbene (100 mg/kg/day) and sacrificed at 0, 2, 6, 10, 12, 24 h (*n* = 4); Part 2: mice treatment with pterostilbene (100 mg/kg/day) or exhaustive exercise for 7 days. **(B)** Body weight was measured one time a day (*F* = 1.384, *p* = 0.261). **(C)** Representative photographs of colons from CON, PTE, EX, and EX + PTE. **(D)** Food intake was measured one time a day (*F* = 0.612, *p* = 0.626). **(E)** TNF-α (PTE: *F* = 63.793, *p* < 0.001; EX: *F* = 287.397, *p* < 0.001; interaction: *F* = 4.354, *p* < 0.05), **(F)** IL-1β (χ2 = 28.102, *p* < 0.001), **(G)** IL-6 (PTE: *F* = 40.789, *p* < 0.001; EX: *F* = 209.477, *p* < 0.001; interaction: *F* = 3.625, *p* < 0.05), and **(H)** glucose were measured by ELISA kits immediately (PTE: *F* = 0.774, *p* = 0.384; EX: *F* = 18.084, *p* < 0.001;interaction: *F* = 1.079, *p* = 0.305). **(I)** Representative PAS images of the colon at a magnification of 100×. **(J)** The colon glycogen was measured (χ^2^ = 20.616, *p* < 0.001). Data were expressed as means ± SEM, and the statistical significance was showed using asterisks denote (*p < 0.05; **p < 0.01; ***p < 0.001).

The C57BL/6 mice (7 weeks, male) weighing 20–22 g were obtained from the Laboratory Animal Centre of the Army Medical University (Chongqing, China) and housed in a controlled environment (22–25^°^C, 50–55%). They were provided standard food (D12450B; 10% fat, 70% carbohydrate, 20% protein) and obtained water freely (29). The body weight and food intake of mice were weighed every day. All animal experiments described herein followed the National Research Council Guidelines, approved by the Animal Care and Use Committee of the Army Medical University. The experimental design is shown in Figure 1A and followed the National Research Council Guidelines. Animal experiment 1: the C57BL/6 mice (*n* = 24) were randomly distributed into six groups (*n* = 4/group), gavaged with pterostilbene (100 mg/kg/day) (30) at 9:00 a.m. and sacrificed at 0, 2, 6, 10, 12, and 24 h after intragastric administration, respectively. The contents of small intestine and colon were collected and stored at −80^°^C. Animal experiment 2: mice (*n* = 48) were randomly divided into four groups (*n* = 12/group): control group (CON), pterostilbene group (PTE), exercise group (EX), and exercise with pterostilbene group (EX + PTE). The experiment lasted for 2 weeks. In the first week, mice ran on the treadmill at a speed of 15 m/min for 10 min one time a day and rested for the weekend on a motorized treadmill (SANS Biological Technology, Jiangsu, China). In the second week, referred to Bedford’s method (31, 32), mice in exercise groups ran at the speed of 25 m/min until exhaustion one time a day for 7 days, and the exhaustion statue according to the literature reported (33). The PTE and EX + PTE groups were orally gavaged with pterostilbene (100 mg/kg/day) for 1 week, as previous study reported (30). The PTE was administered 12 h before exercise, and the mice of the EX and EX + PTE groups were sacrificed 1 h after the last exercise session. Finally, the serum, small intestine, cecum, and colon contents were removed and stored at −80^°^C.

### Cell experiment and design

As the literature reported (34), Caco-2 and CCD 841 CoN cell lines, which were used as intestinal epithelium models frequently, were obtained from the Chinese academy of sciences cell bank or American Type Culture Collection (ATCC) (Manassas, VA, United States). The cells were cultured in Dulbecco’s Modified Eagle’s Medium (DMEM) (Gibco, Carlsbad, CA, United States) containing 10% fetal bovine serum and 1% penicillin–streptomycin in a controlled environment (37^°^C, 95% air, 5% CO_2_).

CCK-8 kits: Cells were seeded at an initial density of 1 × 10^5^ cells/well in a 200 μl DMEM medium. After 24 h, cells were exposed to the freshly prepared medium containing PTE (0, 5, 10, 20, 40, 60, 80, and 100 mg/L) or LPS (0, 0.2, 0.4, 0.6, 0.8, 1.0, 2.0, 4.0, 6.0, 8.0, 10.0 μg/ml) for 24 h. Subsequently, we added 10 μl of CCK-8 solution to each well and measured the absorbance at 450 nm.

The scratch test: Cells were seeded at an initial density of 5 × 10^5^ cells/well in 1 ml DMEM medium. After 24 h, the fused monolayer cells were scraped with the tip of a 10 μl sterile pipette and treated with PTE (20 mg/L) or LPS (1 μg/mL). The cells were photographed 0, 12, 24, and 36 h after the scratch.

Quantitative polymerase chain reaction: (1). Cells were seeded at an initial density of 5 × 10^5^ cells/well in a 2 ml DMEM medium. After 24 h, the PTE and LPS + PTE groups were treated with PTE (20 mg/L). The LPS and LPS + PTE groups were treated with LPS (1 μg/ml) 1 h after PTE treatment. (2). Cells were seeded at an initial density of 5 × 10^5^ cells/well in a 2 ml DMEM medium complemented with the components given above and allowed to attach and grow. After 24 h, cells were treated with LPS (1 μg/ml) and collected at 0, 1, 2, 4, 6, 8, and 12 h. The remaining methods refer to qPCR.

### High-performance liquid chromatography assays for pterostilbene

Apparatus: The assay was set up by an integrated HPLC system (Waters 2695 Liquid Chromatograph, Waters, America). The HPLC system contained a Pntulips QS-C18 Plus column (250 × 4.6 mm i.d., 5 μm), and the Empower 2 software (Waters, America) was used to analyze the data and control the system. HPLC assays: According to the previous method (35), the colon contents (20 mg) were mixed with methanol (500 μl), followed by vortex oscillation (20 s), and ultrasonic extraction (80 Hz, 10 min, ≤ 30^°^C, 12 min). Then, the supernatant was taken as the sample detection solution after standing (4^°^C,1 h) and centrifugation (10,000 g,10 min, 4^°^C). The TMS was used as an internal standard (200 ng/ml). The mobile/water phase was performed by gradient transportation of acetonitrile and 0.1% (v/v) formic acid for 12 min at a flow rate of 1.2 ml/min. The UV absorbance at 320 nm was recorded (36). Program setting: column temperature 35^°^C; gradient procedure:(a) 0–4 min, 60% acetonitrile; (b) 4–8 min, 60–90% acetonitrile; (c) 8–12 min, 90% acetonitrile. The system is rebalanced for 10 min before the next injection.

### Biochemical analysis

As the literature reported (37), serum (10 μl), intestinal contents (50–100 mg), and tissues (50 mg) were weighed and dissolved in 500 μl phosphate-buffered saline (PBS), vibrated (5 min), and centrifugated (12,000 g, 4^°^C, 20 min) to collect the liquid supernatant. The inflammatory cytokines (TNF-α, IL-1β, and IL-6), GSH levels of serum, and LPS levels in the supernatant of intestinal contents were detected by ELISA kits (Quanzhou, Ruixing Biological).

### Histological analysis

Eosin (H&E) staining: The intestine tissues were fixed in 4% paraformaldehyde fixative overnight. The tissues were sectioned at 5 μm and stained with hematoxylin–eosin, observed, and photographed by light microscope as previously described (13). Glycogen staining: Paraffin-embedded sections (5 μm) of the small intestine and colon were dyed in schiff periodic acid shiff (PAS) staining solution B (10 min) and PAS staining solution A (25 min) under dark conditions, respectively. Then, sections were stained with PAS staining solution C (30 s) and observed by a light microscope according to the instruction. Immunofluorescence staining: Paraffin-embedded sections (5 μm) of the colon were prepared and incubated with 0.5% TrixonX-100 (1 h, RT) according to a previous study (13). After that, sections were blocked with 2% horse serum (1 h, RT) and against with primary antibodies (Occludin, Proteintech, 1:1,000) (12 h, 4^°^C) and secondary antibodies (FITC, Beyotime, 1:500) (1 h, RT). After being washed with PBS, sections were incubated with DAPI (Sigma,5 μg/ml) (10 min, RT) and visualized with a fluorescence microscope (Olympus, Japan). The ImageJ software was used to measure the mean fluorescence intensity. Transmission electron microscopy: Tissue samples were sealed with 2.5% glutaraldehyde for 24 h, washed, and fixed in osmium solution for 2 h. Tissue was dehydrated, permeabilized, embedded, and sectioned at 60 nm and observed with a JEM-1400 microscope (JEOL, Tokyo, Japan).

### Quantitative polymerase chain reaction

As previously reported (38–40), the PrimeScript RT Reagent Kit (Takara, Japan) and TRIzol reagent (Invitrogen Life Technologies, Grand Island, NY) were used to extract the total RNAs. Then, we reversed transcript mRNA into cDNA by qTower 2.2 real-time PCR system (Analytik Jena, Germany). The oligonucleotide primers were synthesized by Sangon Biotech (Shanghai, China), as listed in Supplementary Table 1.

### Western blot

As the literature described (39), proteins were extracted from the colon, separated by 12% SDS-PAGE, and transferred onto PVDF membranes (Bio-Rad, CA). After that, membranes were blocked with 5% dried skimmed milk (RT,1 h) and were incubated with primary antibodies (24 h, 4^°^C) under rotation following antibodies against ZO-1 (Abcam, 1:1,000), occludin (Proteintech, 1:1,000), claudin 1 (Thermo Fisher Scientific, 1:1,000). Then, we incubated the membranes with a secondary antibody (1 h, RT). Finally, we used the ImageJ software (NIH, MD) for the quantitative analysis.

### Sequencing of the intestinal flora

We used the DNA Stool kit (Beijing, China) to extract the genomic DNA of bacteria according to the instruction. We analyzed the 16S rRNA gene of the DNA sequence on the Illumina MiSeq platform (Illumina, San Diego, CA, United States), performed by QIIME2 software (41).

### Statistical analysis

All experimental data were expressed as means ± SEM, including at least three biological replicates. The details of the statistical analysis were as follows: experiments between two groups were analyzed with Student’s *t*-test. For multiple group comparisons, we used a two-way ANOVA to analyze the factors’ main effect and interaction and the *t*-test for individual effect analysis after two-way ANOVA. The two analyzed factors of animal experiments were PTE and EX, and the two analyzed factors of cell experiments were LPS and PTE. Moreover, we used the non-parametric Kruskal–Wallis tests if the variance was not the same.

Finally, we used the two-way ANOVA to analyze body weight, food intake, TNF-α, IL-1β, IL-6, glucose, glycogen, quantity analysis of WB, immunofluorescence staining, and results of qPCR of four groups. Besides, we used the *t*-test for two groups’ analysis, such as the healing rate of scratch or other experiments which needed the comparison between the two groups. All analyses were performed by SPSS 19.0 (Chicago, IL). A *p* < 0.05 was considered statistically significant. The statistical significance was showed using asterisks denote (*p < 0.05; ^**^p < 0.01; ^***^p < 0.001).

## Results

### Exercise-induced intestinal barrier disruption and inflammation in mice

**FIGURE 2:**
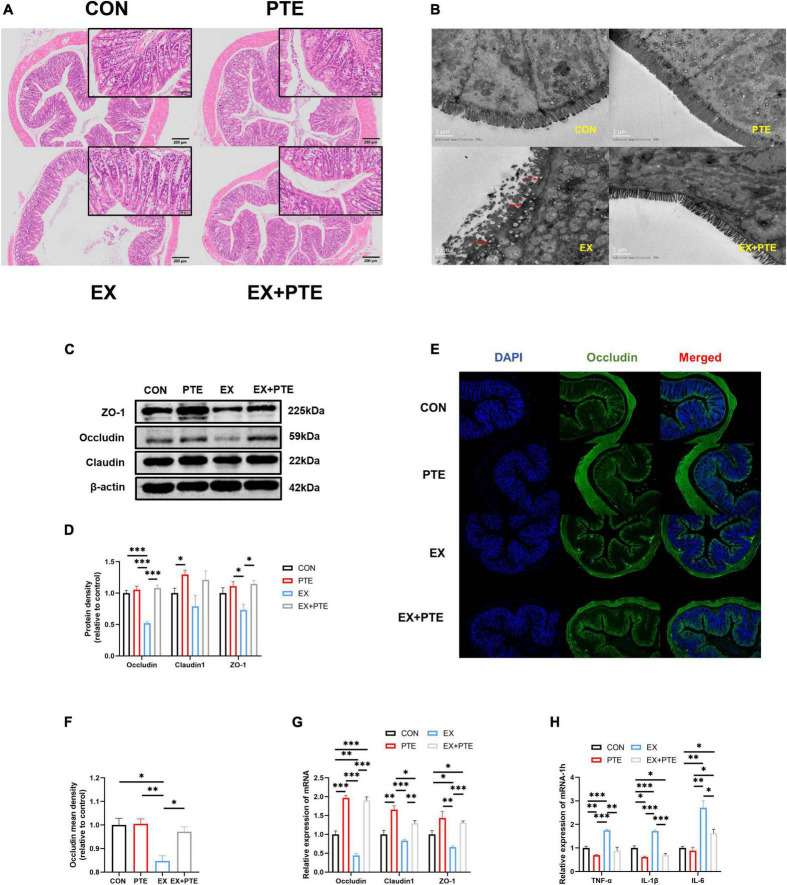
PTE (100 mg/kg/d) inhibited exercise-induced intestinal injury. **(A)** Histological staining of the colon. **(B)** Transmission electron microscopy. **(C,D)** Protein levels of TJ were analyzed by western blotting (occludin: PTE: *F* = 51.611, *p* < 0.001; EX: *F* = 28.061, *p* < 0.001; interaction: *F* = 34.992, *p* < 0.001.Claudin1: PTE: *F* = 8.435, *p* < 0.05; EX: *F* = 1.431, *p* = 0.266; interaction: *F* = 0.269, *p* = 0.618.ZO-1: PTE: *F* = 11.587, *p* < 0.01; EX: *F* = 2.287, *p* = 0.169; interaction: *F* = 3.893, *p* = 0.084). **(E,F)** Colon was observed by the immunofluorescence staining (PTE: *F* = 7.793, *p* < 0.05; EX: *F* = 16.164, *p* < 0.01; interaction: *F* = 6.612, *p* < 0.05). The expression of the intestinal barrier **(G)** (occludin: PTE: *F* = 245.310, *p* < 0.001; EX: *F* = 16.339, *p* < 0.01; interaction: *F* = 9.892, *p* < 0.01. Claudin1: PTE: *F* = 43.313, *p* < 0.001; EX: *F* = 9.654, *p* < 0.01; interaction: *F* = 1.303, *p* = 0.276.ZO-1: PTE: *F* = 26.905, *p* < 0.001; EX: *F* = 4.891, *p* < 0.05; interaction: *F* = 1.061, *p* = 0.323) and the inflammatory factor **(H)** (TNF-α: PTE: *F* = 44.853, *p* < 0.001; EX: *F* = 28.804, *p* < 0.001; interaction: *F* = 10.280, *p* < 0.01.IL-1β: PTE: *F* = 125.588, *p* < 0.001; EX: *F* = 40.647, *p* < 0.001; interaction: *F* = 25.763, *p* < 0.001.IL-6: PTE: *F* = 10.094, *p* < 0.01; EX: *F* = 42.662, *p* < 0.001; interaction: *F* = 6.593, *p* < 0.05) were measured by qPCR. Data were expressed as means ± SEM, and the statistical significance was showed using asterisks denote (*p < 0.05; **p < 0.01; ***p < 0.001).

As shown in Supplementary Figure 1A, sweating and injury of paws in C57BL/6 were significantly observed after running until exhaustive. The body weight (Figure 1B) and food intake (Figure 1D) were the same among the four groups, which indicated that exercise did not change body weight and food intake. There were significant differences in the colonic length (Figure 1C) between the EX group and other groups, and the colonic length of the EX group was shorter than the CON group. After high loading intensity of exercise for 7 days, as previously reported (13), the serum levels of inflammatory cytokines, including TNF-α, IL-1β, and IL-6 in the exercise group, significantly increased (Figures 1E–G). Besides, to confirm the influence on glycogen consumption by exercise, the glucose levels in serum (Figure 1H) and glycogen in intestine tissues of mice were detected by ELISA kits and PAS staining. Moreover, the glycogen level was significantly decreased in the EX group of small intestinal (Supplementary Figures 1B,C) and colon (Figure 1I,J). Histological staining was performed in the small intestine and colon of C57BL/6 mice. The villi of the small intestine (Supplementary Figure 1D) and colon (Figure 2A) were obviously changed with a structural disorder in the EX group compared with the CON group.

To further observe the intestinal damage, transmission electron microscopy was performed. The number of microvilli on the surface of the small intestine (Supplementary Figure 1E) and colon (Figure 2B) villi was reduced and disordered. Furthermore, we analyzed protein levels of the TJ-related genes in the colon tissue by western blotting, and the protein expression of ZO-1, occludin, and claudin1 was decreased significantly in the EX group (Figures 2C,D). Moreover, as observed by the immunofluorescence staining, the mean density of occludin was reduced in the colon of C57BL/6 mice from the EX group (Figures 2E,F). Meanwhile, the mRNA expressions of TJ-related genes were decreased in the EX group (Figure 2G), revealing a disrupted intestinal barrier in the colon induced by the high loading intensity of exercise (13). Besides, the mRNA levels of TNF-α, IL-1β, and IL-6 were significantly increased in the EX group compared with the CON group (Figure 2H), suggesting an inflammatory response in intestinal tissues after exercise. Additionally, the GSH levels of serum were significantly decreased in the EX group (Supplementary Figure 1F), showing that high loading intensity of exercise could induce oxidative stress injury in mice. In brief, the results demonstrated high loading intensity of exercise could cause intestinal barrier disruption and inflammation in C57BL/6 mice.

### Exercise remodeled the intestinal flora related to lipopolysaccharide production

**FIGURE 3:**
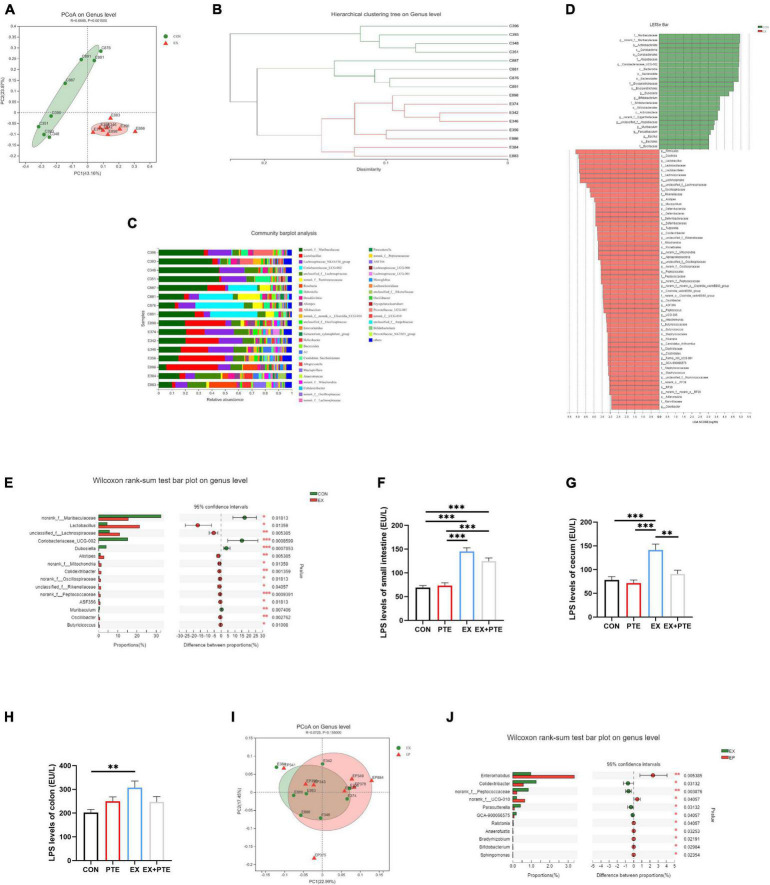
Exercise treatment modified the gut microbiota and induced LPS production. **(A)** Principal coordinate analysis (PCoA) of the β-diversity based on the unweighted UniFrac distance matrix (at the ASV level) of the CON and EX groups. **(B)** Hierarchical cluster analysis. **(C)** The species composition of 16 samples at the genus level, based on the average distance. **(D)** Bar graph of linear discriminant analysis (LDA) scores, showing the biomarker taxa (LDA score of > 2 and a significance of *p* < 0.05 determined by the Wilcoxon signed-rank test). **(E)** The Kruskal–Wallis H test bar plot at the genus level of CON and EX groups. **(F–H)** LPS was detected by ELISA kits of intestinal contents (**F**: PTE: *F* = 1.769, *p* = 0.194; EX: *F* = 104.762, *p* < 0.001; interaction: *F* = 3.724, *p* = 0.064; **G**: χ^2^ = 14.966, *p* < 0.05; **H**: PTE: *F* = 0.091, *p* = 0.765; EX: *F* = 5.766, *p* < 0.05; interaction: *F* = 6.364, *p* < 0.05). **(I)** PCoA of EX and EX + PTE group. **(J)** Kruskal–Wallis H test bar plot at the genus level of EX and EX + PTE groups. Data were expressed as means ± SEM, and the statistical significance was showed using asterisks denote (**p < 0.01; ***p < 0.001).

The cecum contents of mice were collected to investigate exercise’s effect on the intestinal flora. There was no significant difference between CON and EX groups on α-diversity (Supplementary Figures 2A–G), including community richness (Sobs, Chao, and Ace), community diversity (Shannon and Simpson), and community evenness (Simpsoneven and Shannoneven). Moreover, a clear separation was observed by principal coordinate analysis (PCoA) based on unweighted UniFrac distances along the primary ordination axis (PC1), which accounted for 43.16% of the variation (Figure 3A). Based on the average distance, the genus-level species composition of 16 samples was displayed, and hierarchical cluster analysis was performed, indicating that the intestinal flora structure was remodeled by exercise (Figures 3B,C). The analysis of the LEfSe showed that exercise caused an increase in the relative abundance of members from the order Lactobacillales, family Lactobacillaceae, genus Lactobacillus, and genus Alistipes compared with control mice (Figure 3D). Besides, at the genus level, exercise markedly increased the abundance of Lactobacillaceae and Alistipes (Figure 3E), which was related to LPS production as reported (42–44). As accumulating evidence indicated that LPS contributed to intestinal injury, the level of LPS in intestinal contents was measured. The results showed that exercise increased the concentration of LPS significantly (Figures 3F–H) (45, 46). In brief, these results indicated that exercise remodeled the structure of intestinal flora and induced LPS production, which was a major cause of intestinal barrier injury.

### Pterostilbene inhibited intestinal injury induced by exercise

We further detected whether orally garaged with PTE (100 mg/kg/d) in C57BL/6 mice could inhibit intestinal barrier loss induced by the high loading intensity of exercise in the literature (17). We observed that the structural disorder of the small intestine and colon had been improved by histological staining and transmission electron microscopy in the EX + PTE group compared with the EX group (Figures 2A,B and Supplementary Figures 1D,E). Besides, there were significant differences in the gene and protein expression between groups treated with exercise alone or garaged with PTE through a series of indicators (Figures 2C–H). Then, the 16S rRNA gene sequence of cecum content was performed to observe whether PTE could reverse the structure of intestinal flora. At the genus level, there were no significant differences in α-diversity (Supplementary Figures 3A–G), β-diversity (Figure 3I), and the abundance of Lactobacillaceae and Alistipes (Figure 3J) between EX and EX + PTE groups. Besides, based on the average distance, the genus-level species composition was displayed, and hierarchical cluster analysis was performed (Supplementary Figures 3H,I), indicating that PTE could not inhibit LPS production, which was induced by a specific genus. Additionally, the results showed that PTE could improve intestinal barrier loss directly by promoting TJ-related gene expression instead of altering intestinal flora structure.

### High concentration of pterostilbene in intestinal

**FIGURE 4:**
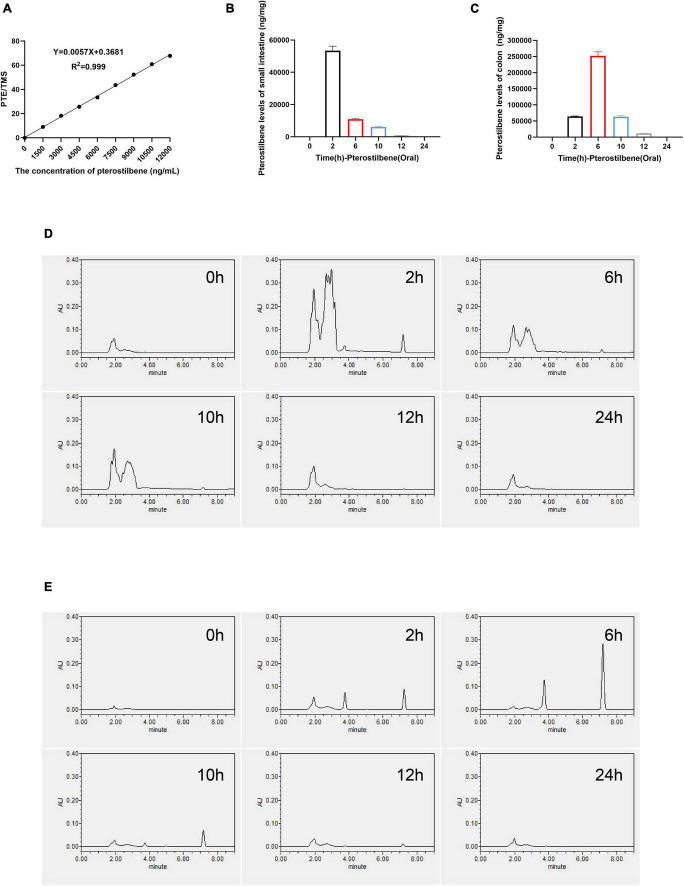
High concentration of pterostilbene in intestinal. **(A)** the calibration curve of pterostilbene obtained by HPLC method at 320 nm in standard solution. **(B,C)** Levels of small intestinal and colon gavaged with pterostilbene (100 mg/kg/day). **(D)** Pterostilbene chromatograms of small intestinal. **(E)** Pterostilbene chromatograms of colon. Data were expressed as means ± SEM.

A convenient and effective high-performance liquid chromatography-ultraviolet (HPLC-UV) method was developed to quantify PTE concentration in intestinal contents, and the TMS was used as an internal standard (Supplementary Figures 4A,B). The peak area ratio (pterostilbene and TMS) was used as the analytical response, and the calibration standards of these concentrations (1,500, 3,000, 4,500, 6,000, 7,500, 9,000, 10,500, and 12,000 ng/ml) were used to generate the calibration curve and assess the linearity (y = 0.0057x + 0.3681, *R*^2^ = 0.999) (Figure 4A and Supplementary Figure 4C). The PTE and TMS eluted from the system at 7.2 and 10.6 min, respectively. Detected by HPLC-UV, the maximum concentration was achieved at 2 and 6 h in small intestinal and colon contents after oral administration of pterostilbene (Figures 4B,C). Besides, an unidentified metabolite that might be rooted in PTE was present at 2–4 min (Figures 4D,E). However, no PTE analytical response was detected in small intestinal and colon contents 12 h after oral administration. In addition, the PTE could keep high concentration in intestinal tissues within 12 h, which might play a crucial role in intestinal barrier repair.

### Pterostilbene preserve tight junction integrity *in vitro*

**FIGURE 5:**
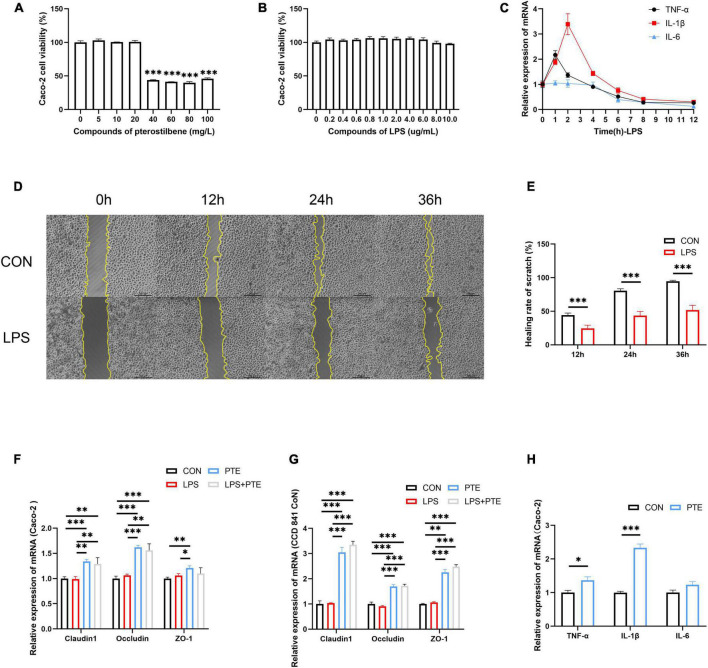
PTE preserve TJ integrity *in vitro*. **(A,B)** The cell viability of Caco-2 cells after pterostilbene (0, 5, 10, 20, 40, 60, 80, and 100 mg/L) or LPS (0, 0.2, 0.4, 0.6, 0.8, 1.0, 2.0, 4.0, 6.0, 8.0, and 10.0 μg/ml) intervention. **(C)** The mRNA expression of inflammatory gene (TNF-α, IL-1β, and IL-6) induced by LPS (1 mg/ml) at 1, 2, 4, 6, 8, and 12 h. **(D,E)** The healing rate of LPS-treated cells by the scratch test. **(F)** The expression of TJ-related gene after PTE (20 mg/L) treatment by qPCR analysis in Caco-2 cells (claudin1:LPS: *F* = 0.398, *p* = 0.540; PTE: *F* = 44.602, *p* < 0.001; interaction: *F* = 0.174, *p* = 0.684. occludin: χ2 = 1.333, *p* = 0.248.ZO-1: LPS: *F* = 0.380, *p* = 0.549; PTE: *F* = 8.755, *p* < 0.05; interaction: *F* = 4.126, *p* = 0.065), **(G)** CCD 841 CoN cells (claudin1: LPS: *F* = 1.930, *p* = 0.190; PTE: *F* = 327.213, *p* < 0.001; interaction: *F* = 1.196, *p* = 0.296. occludin: LPS: *F* = 0.283, *p* = 0.604; PTE: *F* = 166.214, *p* < 0.001; interaction: *F* = 1.125, *p* = 0.310.ZO-1: χ2 = 2.083, *p* = 0.149). **(H)** the expression of inflammatory gene in Caco-2 cell between CON and PTE (20 mg/L). Data were expressed as means ± SEM, and the statistical significance was showed using asterisks denote (*p < 0.05; **p < 0.01; ***p < 0.001).

Cell experiments were performed to verify PTE’s potential effects against intestinal barrier injury *in vitro*. The cell viability of Caco-2 did not change in the presence of a chosen concentration of PTE (0–20 mg/L) and LPS (0–10 mg/L) (Figures 5A,B). Reverse transcription polymerase chain reaction (RT-PCR) analysis showed that LPS-induced inflammatory response occurred in Caco-2 cells (Figure 5C) at early stages (1–3 h), which were subsequently decreased after 6 h, further confirming the results of animal experiments in Figures 1E–G. Besides, there were no significant differences in TJ-related genes (occludin, claudin-1, ZO-1) in Caco-2 cells at different points (Supplementary Figure 4D), indicating that LPS could not affect TJ-related gene expression.

Additionally, the scratch test showed that the healing rate of LPS-treated cells (24.5 ± 0.7%;43.5 ± 0.9%; 51.8 ± 1.0%) was significantly decreased than control (44.2 ± 0.4%;80.6 ± 0.4%; 94.6 ± 0.2%) at 12, 24, and 36 h (Figures 5D,E). The control experiments were carried out to investigate the potential effects of PTE (20 mg/L,12 h) after LPS (1 mg/L, 1 h) exposure. There were significant improvements in TJ-related genes after PTE treatment compared with other groups by RT-PCR analysis in Caco-2 cells or CCD 841 CoN cells (Figures 5F,G). However, the expression of inflammatory factor genes (TNF-α, IL-1β, and IL-6) was increased obviously after PTE (20 mg/L) intervention in Caco-2 cells (Figure 5H) compared with CCD 841 CoN cells (Supplementary Figure 4E), owing to the anticancer effect of PTE. In addition, treatment with PTE could induce the expression of TJ-related genes.

## Discussion

Many studies have proved that exercise is beneficial for health, but the high loading intensity of exercise is harmful to human health. It has been well established that substantial exercise might induce GIS and decrease exercise performance for the athlete population (7, 47). Besides, a common feature of GIS is the altered intestinal permeability (48). The high loading intensity of exercise might affect the downregulation of the TJ-related genes to increase intestinal permeability. Some studies found that the loss of barrier integrity contributes to inflammatory bowel disease and other metabolic diseases (49). The barrier integrity could prevent LPS produced by gut microbiota from transferring into the serum. In this study, we found that the high loading intensity of exercise-induced GIS in C57BL/6 mice mode was related to the disrupted intestinal barrier integrity (4). However, the potential mechanisms remained to be elucidated.

A previous study showed that change in intestinal flora composition was related to metabolism disturbance (50), which might damage intestinal integrity (51). Gut-derived LPS, which is induced by the increased proportions of harmful microorganisms (such as Alistipes) (42), plays a crucial role in causing intestinal inflammatory responses (52). In this study, we found that the intestinal flora composition of C57BL/6 mice was altered by the high loading intensity of exercise, which increased the abundance of Alistipes and caused a high concentration of LPS in contents.

The PTE has been largely investigated for its’ anti-inflammation (53), anticancer (54), antiobesity (55), and antifibrosis effects (56) in the past decade. The PTE reduces blood pressure in adults at 250 mg/day doses (57). Besides, the PTE at 100 mg/kg/day doses could preserve the exercise endurance of mice subjected to sleep restriction in mice (30). PTE at 50 mg/kg/day doses could promote skeletal muscle adaptations to exercise training in rats (58). However, the effect of high doses of PTE on athletes is still unknown. In this study, we aimed to investigate the role of PTE in the occurrence of intestinal barrier repair to prevent perturbed intestinal function followed by high load intensity exercise. The PTE has a significant interest in preventing TJ integrity *via* promoting the expression of intestinal epithelial TJ molecules. Furthermore, some studies found that PTE protected the intestinal epithelial barrier through the NF-κBMLCK/p-MLC signal pathway in mice (27). Our results showed that high load intensity of exercise could induce disrupted intestinal barrier integrity and inflammation response in the C57BL/6 mice running model, which might be due to the LPS produced by intestinal flora (59). Otherwise, oral administration of PTE could significantly prevent intestinal barrier damage by improving the expression of the TJ-related genes.

Unexpected *in vitro* experiment, we found that there was no obvious change in the expression of the TJ gene after LPS treatment, showing that LPS-induced intestinal barrier disruption might be related to the immune microenvironment of intestinal and deserves further study (Supplementary Figure 4D). Overall, this is the first report on PTE improving intestinal barrier integrity disrupted by the high loading intensity of the exercise *via* promoting the expression of intestinal epithelial TJ molecules. However, the potential mechanism is still unclear, which will also be a part of our follow-up study in the future. Besides, there were some limitations in our study. We found that PTE (100 mg/kg/d) could not reverse the intestinal flora structure induced by high load intensity exercise, which indicated that PTE could not prevent the dysbiosis of intestinal flora composition. Moreover, our study only obtained the results from animal and cell experiments, which could be different in humans. Therefore, we will further observe the effect of PTE on athletes, which will also be a part of our follow-up study in the future.

## Conclusion

**FIGURE 6:**
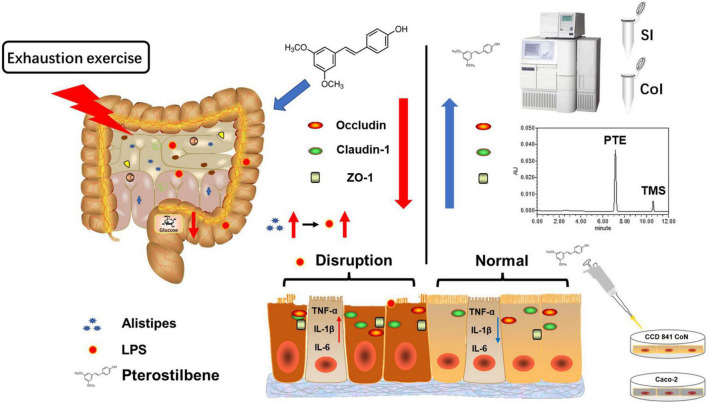
Schematic summary of the results. High loading intensity of exercise disrupted the intestinal epithelial barrier through remodeling the gut flora related to LPS production. Pterostilbene attenuated exercise-induced intestinal barrier injury by improving the expression of the TJ-related genes and suppressing inflammation.

In summary, high load intensity of exercise would affect intestinal permeability caused by LPS, which might be related to altering intestinal flora structure in the cecum (Figure 6). Certain dietary supplements might contribute to the prevention of injury induced by exercise (60). Therefore, we found PTE emerging as a promising candidate for a new generation of sports nutrition supplements for athletes.

## Data availability statement

The original data supporting the conclusions of this article will be provided by the corresponding author, without undue reservation. The raw data of 16S rRNA sequencing reported in this manuscript are deposited in the NCBI database (accession number PRJNA846737). Available online at: https://www.ncbi.nlm.nih.gov/bioproject/846737.

## Ethics statement

All animal experiments described herein followed the National Research Council Guidelines, approved by the Animal Care and Use Committee of the Army Medical University.

## Author contributions

LZ designed the experiments and drafted the manuscript. GT and LZ collected the samples and performed the experiments. LH assisted in running training and aided in analyzing the data. MZ contributed to technical support. MM, LY, and JZ obtained funding and provided many suggestions on the experiments and the article. All authors contributed to the article and approved the submitted final manuscript.

## Abbreviations

PTE: pterostilbene
LPS: lipopolysaccharide
ATCC: American Type Culture Collection
DMEM: Dulbecco’s Modified Eagle’s Medium
TMS: 3,5,4’-trimethoxy-trans-stilbene
DAPI: 4’,6-diamidino-2-phenylindole
GIS: gastrointestinal syndrome
TJ: tight junction
PCoA: principal coordinate analysis
PC1: primary ordination axis.
