# Supplementary material for: Pterostilbene attenuates intestinal epithelial barrier loss induced by high loading intensity of exercise
Source: Front Nutr. 2022 Aug 4;9:965180. doi: 10.3389/fnut.2022.965180 (PMC9386544; doi:10.3389/fnut.2022.965180)
Supplement: Supplementary file 1 [file Data_Sheet_1.docx]

***Supplementary Material***

**1.1** **Supplementary Tables**

**
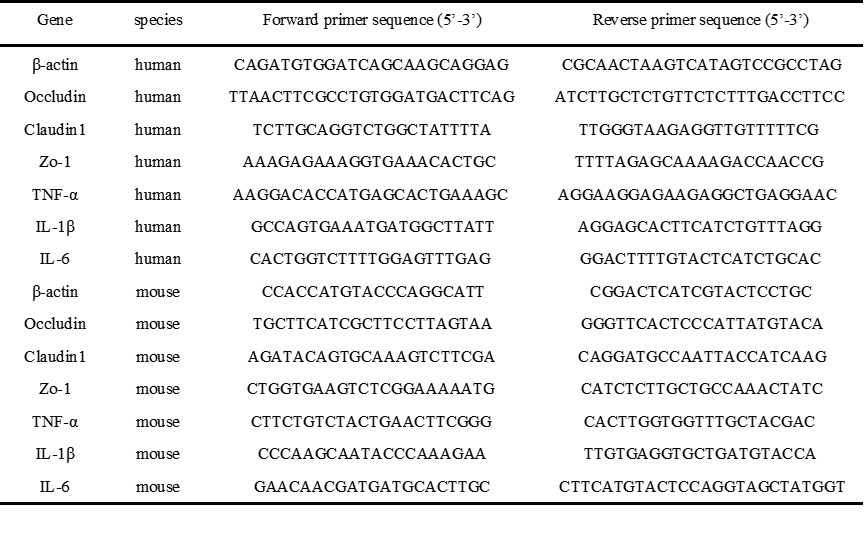
**

**Table 1. Primers used for RT-PCR.**

**1.2 Supplementary Figures**

**1.2.1 Supplementary Figure 1**

**
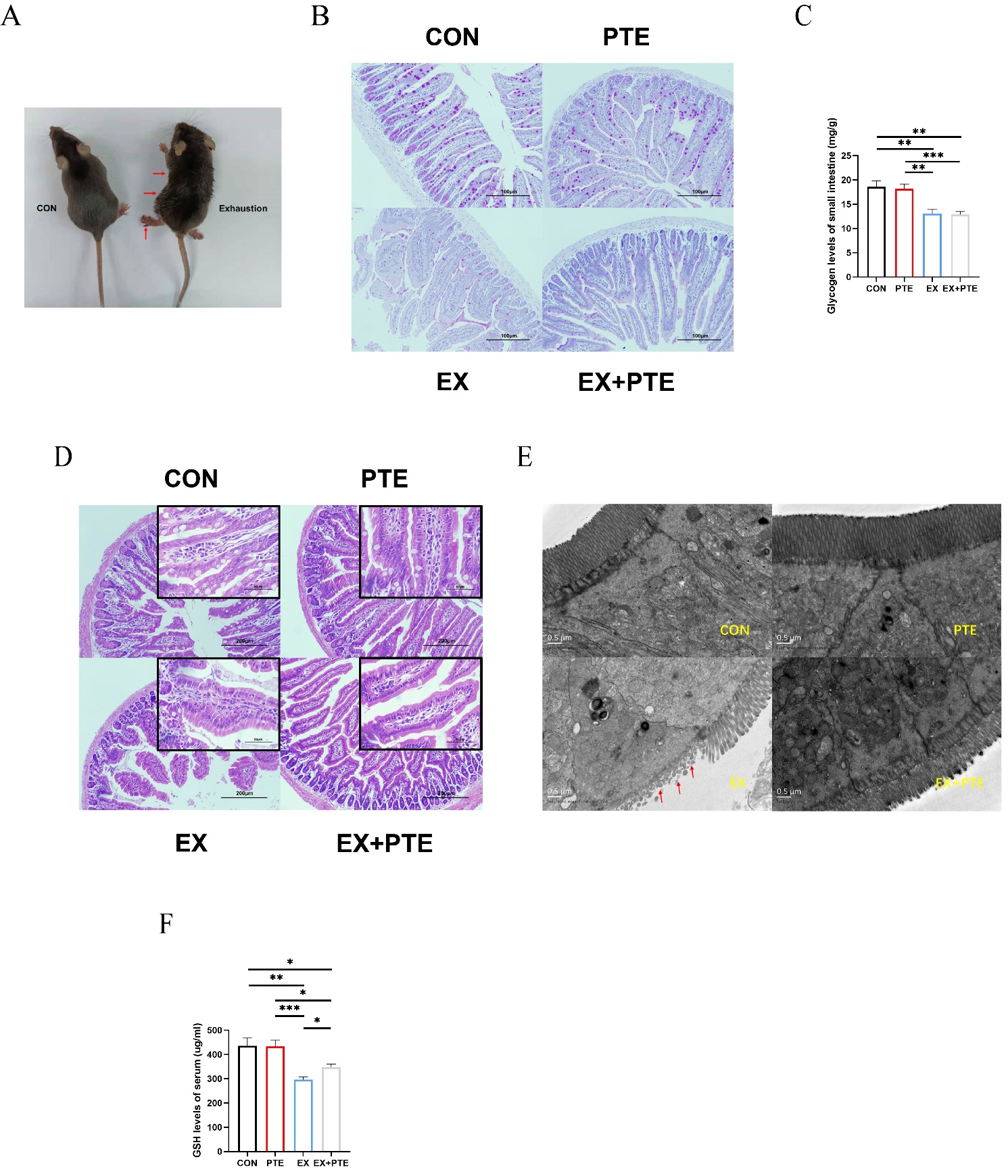
**

**Supplementary Figure 1.** Exercise-induced intestinal barrier disruption of the small intestine in C57BL/6 mice. (A) The status of C57BL/6 mice after running on the treadmill. (B-C) Representative PAS images of the small intestine at a magnification of 100× and detected by ELISA kits (PTE: F=0.094, P=0.762; EX: F=31.432, P<0.001; interaction: F=0.010, P=0.921). (D) Histological staining of the small intestine at a magnification of 200×. (E) Transmission electron microscopy of the small intestine at a magnification of 25000×. (F) GSH levels of serum (χ2 =16.241, P<0.05). Data were expressed as means ± SEM, the statistical significance was showed using asterisks denote (∗p < 0.05; ∗∗p < 0.01; ∗∗∗p < 0.001).

**1.2.2 Supplementary Figure 2**

**Supplementary Figure 2.** The α-diversity of intestinal flora between CON and EX group. (A) Sobs index. (B) Chao index. (C) Ace index. (D) Shannon index. (E) Simpson index. (F) Simpsoneven index. (G) Shannoneven index.

**1.2.3 Supplementary Figure 3**

**Supplementary Figure 3.** The α-diversity of intestinal flora between EX and EX+PTE group. (A) Sobs index. (B) Chao index. (C) Ace index. (D) Shannon index. (E) Simpson index. (F) Simpsoneven index. (G) Shannoneven index. (H) hierarchical cluster analysis. (I) Species composition at the genus level.

**1.2.4 Supplementary Figure 4**


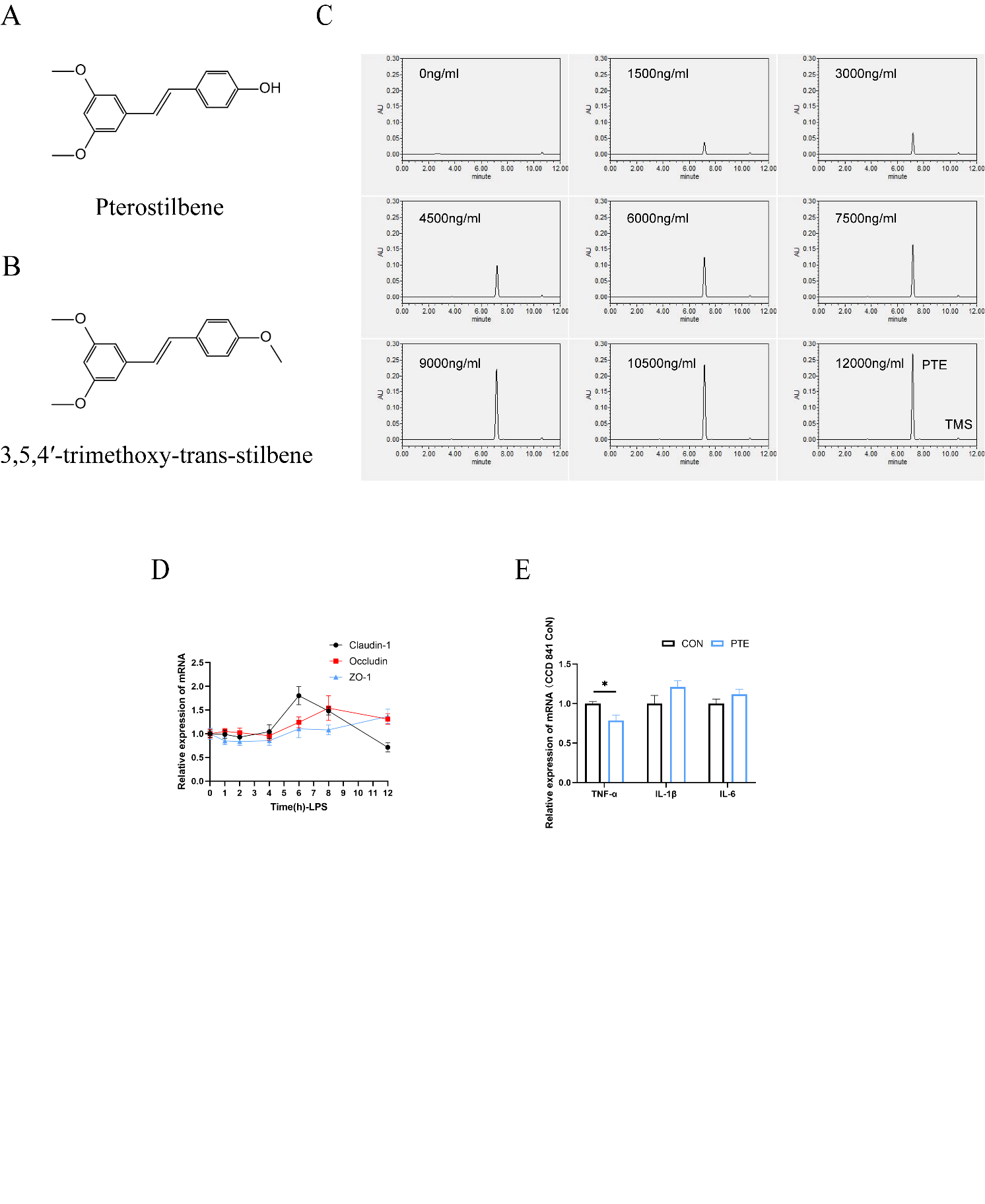


**Supplementary Figure 4.** The calibration curve of PTE. (A) Chemical structures of pterostilbene. (B) Chemical structures of TMS. (C) The calibration curve of PTE. (D) the mRNA expression of TJ related gene (ZO-1, Occludin, Claudin1) induced by LPS (1mg/ml) at 1,2,4,6,8,12h. (E) the mRNA expression of the Inflammatory gene (TNF-α, IL-1β, IL-6) in CCD 841 CoN cells between CON and PTE (20mg/L). Data were expressed as means ± SEM, the statistical significance was showed using asterisks denote (∗p < 0.05; ∗∗p < 0.01; ∗∗∗p < 0.001).
